# Epidemiological model based periodic intervention policies for COVID-19 mitigation in the United Kingdom

**DOI:** 10.1038/s41598-022-19630-6

**Published:** 2022-09-19

**Authors:** Gianmario Rinaldi, Prathyush P. Menon, Antonella Ferrara, W. David Strain, Christopher Edwards

**Affiliations:** 1 The Centre for Future Clean Mobility, Department of Engineering, Faculty of Environment, Science and Economy, University of Exeter, Exeter, EX5 2GD United Kingdom; 2 EPSRC Hub for Quantitative Modelling in Healthcare, University of Exeter, Exeter, EX4 4QF United Kingdom; 3 Department of Electrical, Computer and Biomedical Engineering, University of Pavia, 27100 Pavia, Italy; 4 Medical School, University of Exeter, Exeter, EX1 2LU United Kingdom; 5 Department of Engineering, Faculty of Environment, Science and Economy, University of Exeter, Exeter, EX4 4QF United Kingdom

**Keywords:** Electrical and electronic engineering, Engineering, Public health

## Abstract

As the UK, together with numerous countries in the world, moves towards a new phase of the COVID-19 pandemic, there is a need to be able to predict trends in sufficient time to limit the pressure faced by the National Health Service (NHS) and maintain low hospitalisation levels. In this study, we explore the use of an epidemiological compartmental model to devise a periodic adaptive suppression/intervention policy to alleviate the pressure on the NHS. The proposed model facilitates the understanding of the progression of the specific stages of COVID-19 in communities in the UK including: the susceptible population, the infected population, the hospitalised population, the recovered population, the deceased population, and the vaccinated population. We identify the parameters of the model by relying on past data within the period from 1 October 2020 to 1 June 2021. We use the total number of hospitalised patients and the fraction of those infected who are being admitted to hospital to develop adaptive policies: these modulate the recommended level of social restriction measures and realisable vaccination target adjustments. The analysis over the period 1 October 2020 to 1 June 2021 demonstrates our periodic adaptive policies have the potential to reduce the hospitalisation by 58% on average per month. In a further prospective analysis over the period August 2021 to May 2022, we analyse several future scenarios, characterised by the relaxation of restrictions, the vaccination ineffectiveness and the gradual decay of the vaccination-induced immunity within the population. In addition, we simulate the surge of plausible variants characterised by an higher transmission rate. In such scenarios, we show that our periodic intervention is effective and able to maintain the hospitalisation rate to a manageable level.

The Coronavirus Disease-2019 (COVID-19, caused by the virus novel Severe Acute Respiratory Syndrome Coronavirus 2: nSARS-CoV-2^1^), originally identified in Wuhan, China, in late 2019^2^, has disrupted the hospital networks in the UK and around the world. The initial lack of appropriate non-pharmaceutical interventions, prophylactic therapy and acute treatments led to high numbers of admissions to hospitals. Since March 2020 to date, nearly a million people have been hospitalised with COVID-19 in the UK, with a peak bed occupancy of 39,254 (i.e., more than 33% of the bed capacity) recorded on 18 January 2021^3–5^. Whilst the healthcare expenditure has been increased by 12.8% of GDP in the UK in 2020, this was also responsible for a significant delay in elective and non-elective care (a drop of nearly 20% in non-COVID-19 admissions^6^), postponed appointments of outpatients and limited the ability for Primary Care to perform face to face consultations. During the first wave of the pandemic, the COVID-19 outbreak also caused an unprecedented excess of mortality in care homes^7^. These elements give a perspicuous explanation of the wider pressure the NHS has been subjected to beyond the hospitalised patients. Wide-spread non-pharmacological interventions, social restrictions and lock-downs were imposed to try to slow down and halt the spread of the Coronavirus^8^.

Additionally, there has been an unprecedented cancellation and postponement of non-COVID-19 treatments including in some cases cancer treatments to prioritise the support of COVID-19 patients.

The bed capacity in the NHS hospitals was consequently rearranged to better cope with the extraordinary pandemic challenges and new so-called Nightingale hospitals were built^4^. The repercussions of these interventions, in England and Wales, resulted in 607,922 recorded deaths in 2020, 14% higher than the national average over previous years, and the most excess deaths since World War Two^9^. This grave reality caused by COVID-19 called for the need to manage the NHS hospital admissions more efficiently and possibly automatically^10^, whilst also being better prepared for further waves of the current or future pandemics^11,12^.

**Figure 1:**
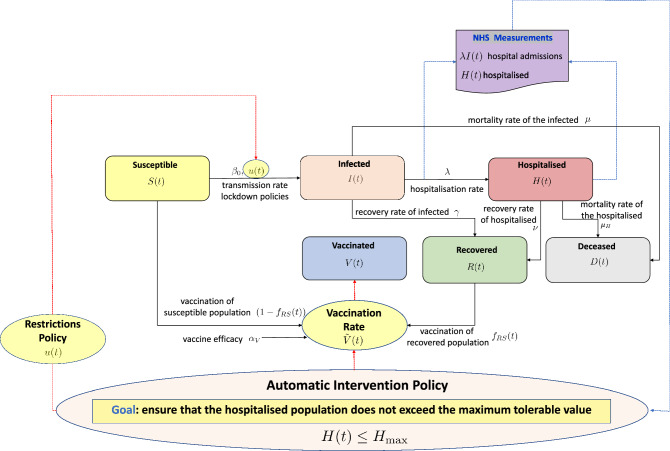
A schematic of the adopted compartmental SIHRD-V epidemiological model The directions of the arrows indicate a positive influence on the considered signal. The blue dashed line indicates the NHS measurements, whilst the red dashed lines the interventions policies.

**Figure 2:**
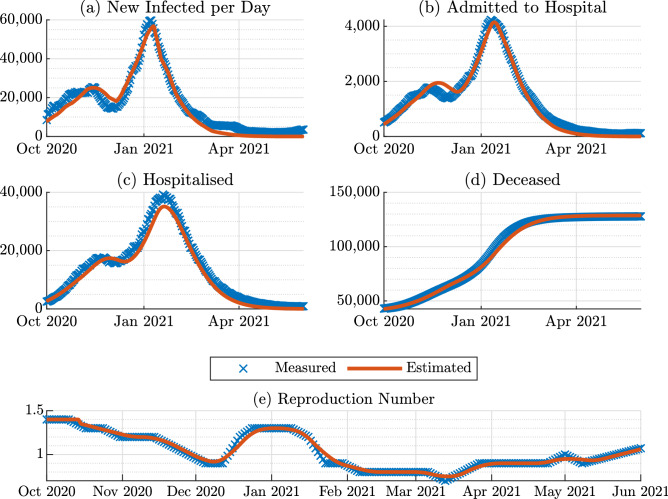
Comparison between measured data and estimation from the COVID-19 model identification: (**a**) Time histories of the people tested positive, (**b**) patients admitted to hospital, (**c**) patients in hospital, (**d**) deceased population, (**e**) and the reproduction number over ${\mathcal {R}}(t)$ the period October 2020-June 2021.

From December 2020, thanks to the unprecedented efforts of medical research, mass vaccination campaigns started worldwide and in the UK^3,13^, raising the hope that the COVID-19 disease could finally be beaten via immunisation of the population. Multiple types of vaccines have been approved by national and international health organisations in order to reduce hospitalisations and death due to the disease. However, they have not halted the transmission of the virus and hospitalisations^14^. Several studies have also shown how the freedom of movement of people can potentially lead to a surge of infections^15,16^ .

In the meantime, there has been a rapid spread of new COVID-19 variants, such as the Alpha Variant (B1.1.7, first identified in Kent) in December 2020, the Gamma Variant (P.1, first identified in Brazil) and the Delta Variant (B1.316.2, first identified in India) during the Spring of 2021^17^. Furthermore, the vaccination campaign was also characterised by vaccine shortages and difficulties in conceiving an efficient vaccination plan^18^. These issues revealed that a blended approach based on both pharmaceutical interventions (i.e. the vaccination campaigns), and non-pharmaceutical interventions (i.e social restrictions and lockdowns policies) was required to slow down the spread of the virus so as to bring pressure on the NHS back within the limits of sustainability^19^.

Since the COVID-19 disease started spreading, the mathematical modelling has been utilised as a front-line tool to decide the level of non-pharmaceutical interventions in the UK^20^. Mathematical modelling entails developing a set of equations whose solutions mimic the evolution of a real process. This approach can be effectively used to describe and understand the spread of a viral disease such as Coronavirus. Different solutions have been proposed in the literature to identify suitable models to reproduce the real spread of the disease^21–23^. The mathematical model can be refined and tuned by utilising available time-series data published by Public Health England - such as the number of hospitalised patients from the population, the number of new infections, and the number of deceased.

The COVID-19 modelling approach proposed in this paper partitions the population into compartments, such as the susceptible population (*S*(*t*)), the infected population (*I*(*t*)), the hospitalised population (*H*(*t*)), the recovered population (*R*(*t*)), the deceased population (*D*(*t*)), and the vaccinated population (*V*(*t*)). Conventionally, the mathematical models are denoted via the acronyms composed of the initials of the compartments adopted. For example, the well-known SIR model^24^ includes only the susceptible, infected and recovered population, whilst the SIHRD model^25^ includes also the hospitalised, the recovered and the deceased ones. The model, by means of simulation, determines the evolution over time of the number of people belonging to each compartment, and it receives as inputs the lockdown (non-pharmaceutical) interventions policy, and the vaccination rate policy. The model is also characterised by key-parameters (which are usually unknown and time-varying), namely the transmission rate, the hospitalisation rate, the recovery rate and the mortality rate of the COVID-19 disease^26^.

In this class of mathematical models, three key-parameters are typically utilised to describe the spread of a viral disease such as COVID-19: the Basic Reproduction Number (${\mathcal {R}}_0$), the Reproduction Number (${\mathcal {R}}(t)$), and the Current Reproduction Number (${\mathcal {R}}_c(t)$). The Basic Reproduction Number is a fixed parameter and it is the expected number of infected people generated by a single infected individual in a completely susceptible population^27^. The Basic Reproduction Number is estimated to be around 3 for the first strain of Coronavirus originated in Wuhan, China, whilst it is estimated to be around 7 for the Delta Variant^28^. The Reproduction Number is instead a time-varying parameter, and it is defined as the average number of new infections caused by a single infected individual (in a population only partially susceptible)^29^. The Current Reproduction number is obtained multiplying the Reproduction Number by the ratio between the current number of susceptible individuals and the total population^13^. Whenever the Current Reproduction Number is greater than one, the number of infected individual increases over time. It is important to note that the Reproduction Number and the Current Reproduction Number are predominantly influenced by the non-pharmacological interventions employed^29^.

In the available published research, the use of mathematical models for COVID-19 has been typically focused on predicting the pandemic evolution under different scenarios. For example, a recent study^13^ shows the impact of different vaccination rates along with the surge of new Coronavirus variants in Italy. This study revealed the impact of pre-defined intervention policies on the hospitalisation occupancy. Mathematical models have also been adopted to predict the bed occupancy in the South West of England^30^.

Research on the design of automatic model-driven periodic intervention policies to mitigate the spread of COVID-19 disease is still in its infancy. The key-idea of such a line of research is to guide automatically the intervention policies (such as target vaccination rates and the extent of social restrictions) over time to avoid unbearable levels of pressure on the NHS. Only a few studies have already been published. For instance, the use of a SIHRD mathematical model for COVID-19 spread has shown that it is possible to maintain the infected population number under a prescribed level^31^, also considering the United States topology^25^. A recent study has demonstrated that a periodic scheme, which is composed of short lockdown periods followed by an easing of restrictions, has the potential to slow down the Coronavirus spread^32^.

Distinctively, in our study, we propose an original model-based automatic and periodic adaptive intervention policy, which aims to keep the hospitalised population below a chosen threshold level, thus alleviating the pressure on the NHS (note that the attributes “periodic adaptive” are here to be understood in the sense that the intervention policy is adapted to the current situation with a certain temporal frequency). We adopt an original SIHRD-like model accounting also for the vaccination campaign, and we identify the relevant parameters relying on open-data relating to the UK. We undertake two tests of our scheme:

(i)

*Retrospective Test:* We undertake a comparison during the time period October 2020-June 2021 between the model-free intervention policy, which was applied in reality in the UK, and our proposal. In this study, it is shown that our policy would have been able to keep the pressure on the NHS to a more manageable level.

(ii)

*Prospective Test:* We focus on the period August 2021-May 2022, in which we simulate the appearances of new COVID-19 variants. These variants are characterised by a higher transmission rate and a higher hospitalisation rate. We also model the loss of COVID-19 vaccination-induced immunity within the population over time. Our model-based automatic design of policies for pharmacological and non-pharmacological interventions are demonstrated to be successful in limiting hospitalisation in the wake of plausible COVID-19 variants and the loss of vaccination immunity in the future.

To the best of our knowledge, the use of an automatic approach to keep the hospitalised population under a prescribed (possibly time-varying) level with application to the COVID-19 pandemic has never been proposed before. Our finding has the potential to be utilised as a tool to adapt the NHS hospital capacity over time, and the underlying principle can be extended to other viral diseases.

## Results

### COVID-19 mathematical model

In this paper, we propose an extended version of the SIHRD model which accounts for periodic interventions in terms of the restrictions and the vaccination campaign in the UK. We name this model SIHRD-V, the schematic of which is shown in Fig. 1. We make use of the publicly available pandemic data on new admissions to hospital, the vaccination rate and the total hospitalisation per day^3,19^ during 1 October 2020 and 1 June 2021 to identify the different parameters of the model. Following the method of nonlinear grey-box identification^33^, the rate parameters of the SIHRD-V model corresponding to recovery of the infected (0.1150), hospitalisation (0.0103), recovery of the hospitalised (0.0954) and mortality of the infected (0.0020) and the hospitalised (0.010) are determined. A comparison between the measured and the simulated data, shown in Fig. 2, primarily illustrates the quality of our model and the identification of its rate parameters.

Existing model based approaches^25,31^ rely on the number of infected population, which is more difficult to acquire^34^ and often underestimates the actual value of the infected population^35^. In contrast, we use the total hospitalised population and the infected population being admitted to hospital each day, which are accurately gathered on a daily basis via the NHS records^3^. For given initial conditions concerning the hospitalised, the deceased and the recovered population, it is possible to alter different parameters, such the as infection rate of the virus and the vaccine efficiency, of the original SIHRD-V model and determine the evolution of the states of the COVID-19 pandemic in the UK and gauge their impact on society.

#### Intervention methodology

We design a model-driven intervention scheme, which aims to automatically select the value of the restrictions policy and the vaccination rate, to maintain the number of the hospitalised population below a defined value. The objective is achieved by relying on only the two aforementioned measured quantities. The fundamental principle, which is further detailed in the Methods section of this paper, is based on a so-called “relays” architecture, as the restrictions policy automatically switches amongst six possible values according to the present number of hospitalised people.

In order to ensure the practical feasibility of our approach, the periodic interventions are updated at a frequency of 2 weeks. More precisely, our strategy chooses which one of the six possible levels of restrictions to put in place for the upcoming two weeks. We choose these levels relying on the past evolution of the restrictions adopted in the UK over the period October 2020-June 2021. The selected levels with an increasing order of magnitude are: (1) No restrictions in place other than recommendations about crowded places and personal hygiene (0.66); (2) A low level of restrictions, with certain limits on large events and social gatherings (0.77); (3) A medium level of restrictions with limits on indoor social gatherings (0.82); (4) An enhanced level of restrictions, with no indoor social gathering (0.84); (5) A high level of restrictions, with closures of all hospitality sectors (0.86); (6) A national lockdown, involving the closure of all non-essential shops and stay-at-home instructions (0.88).

**Figure 3:**
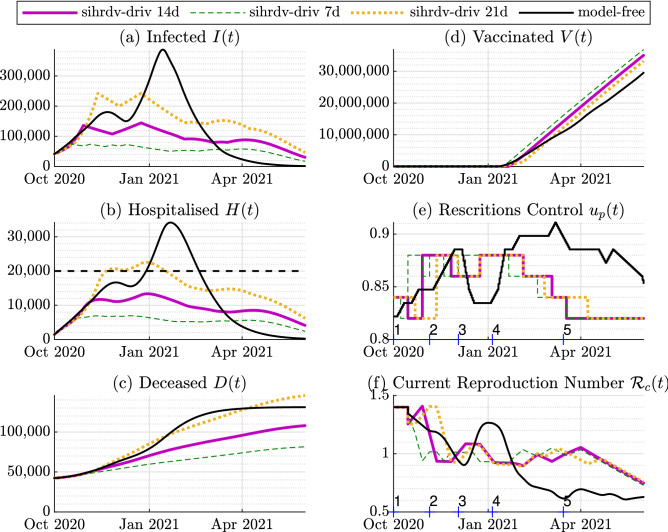
Time histories of the retrospective analysis: 1 October 2020-1 June 2021. The purple line represents our SIHRD-V model-driven results with a policy update of 14 days (sihrdv-driv 14d). We also include results with a policy update of 7 days (the green dashed line named sihrdv-driv 7d), and of 21 days the (orange dotted line named sihrdv-driv 21d). The black line represents the actual intervention policy followed in the UK. The black dashed line is desired upper limit for the hospitalisation (equal to 20,000). The hospitalised population remains below 20,000 if our intervention strategy is used. The numbers $1,2,\dots ,5$ on the horizontal axis in Sub-Figures (**e**) and (**f**) represent the instants when adjustments are taken in the model-free setup.

**Figure 4:**
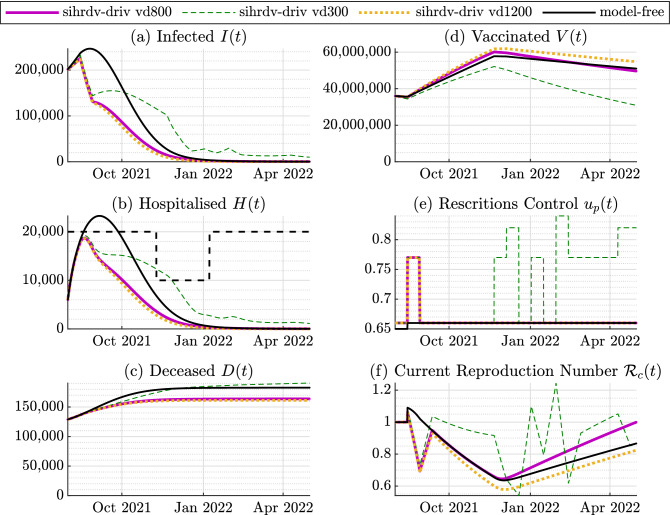
Time histories of the perspective test: August 2021-May 2022. No new COVID-19 variants. The basic reproduction number is kept equal to ${\mathcal {R}}_0=7$ (Delta Variant). The purple line named sihrd-driv vd800 represents our SIHRD-V model-driven results with a vaccination immunity decay time constant of 800 days. We also include the SIHRD-V model-driven results with a vaccination immunity decay time constant of 300 days (the green dashed line named sihrd-driv vd300), and of 1200 days (the orange dotted line named sihrd-driv vd1200). The black line represents the model-free intervention policy, i.e. without any restrictions adjustment. The black dashed line represents the chosen time-varying threshold for the hospitalised population. The hospitalised population remains below the maximum accepted time-varying threshold with the use of our intervention strategy.

**Figure 5:**
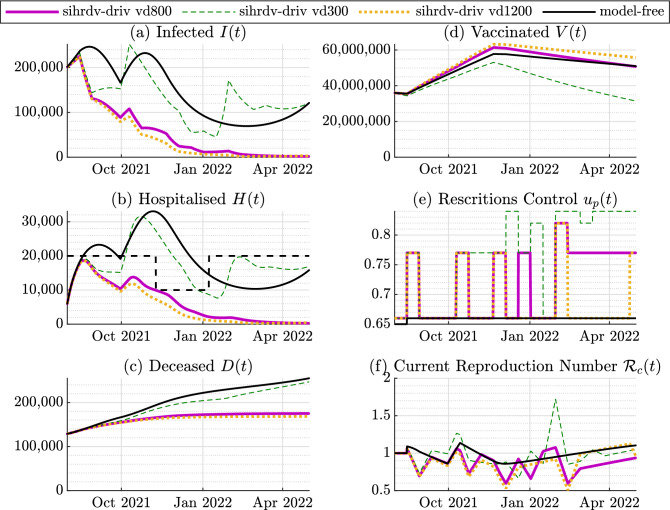
Perspective Test August 2021-May 2022. A new variant with ${\mathcal {R}}_0=10$ appears in October 2021. The purple line named sihrd-driv vd800 represents our SIHRD-V model-driven results with a vaccination immunity decay time constant of 800 days. We also include the SIHRD-V model-driven results with a vaccination immunity decay time constant of 300 days (the green dashed line named sihrd-driv vd300), and of 1200 days (the orange dotted line named sihrd-driv vd1200). The black line represents the model-free intervention policy, i.e. without any restrictions adjustment. The black dashed line represents the chosen time-varying threshold for the hospitalised population. The hospitalised population remains below the maximum accepted time-varying threshold with the use of our intervention strategy.

Accordingly, the realisable vaccination target rate is adjusted too (as explained in the Methods section of this paper). Furthermore, we select a nominal value for the threshold equal to 20,000, which is approximately 17% of the total bed capacity of the NHS^4^. If the threshold value is adjusted over time, our scheme is capable of readjusting the level of restriction also. We introduce a performance metric which captures whenever the hospitalised population passes its threshold. The greater the value of the metric, the greater the extent to which the hospitalised population passes its nominal threshold.

### Retrospective test

The intervention policy adopted in the UK during October 2020 and June 2021 is shown in black in Fig. 3. The peak bed occupancy by COVID-19 patients in hospitals was over 39,000, which corresponded to 33% of the total beds available in all the hospitals across the UK. In the retrospective test considered here, we investigate possible alterations to restrictions such that the hospitalisation level is maintained below a threshold of 20,000. We simulate the response of the identified SIHRD-V model in two setups.

The first one concerns the application of the proposed automatic periodic intervention policy, driven by the SIHRD-V model. The second one is what was actually adopted in the UK between October 2020 and June 2021. The same was characterised by five main restrictions updates imposed by the UK government^5,19^:

1. The three-tier alerts system in the period 1 October 2020-5 November 2020 imposed local social restrictions in specific geographical areas with COVID-19 outbreaks. As shown in Fig. 3e, this intervention smoothly reduced the current reproduction number. However, the surge in infections and hospitalisation was not halted.
2. A 4-weeks national lockdown from 5 November 2020 to 2 December 2020. It can be seen from Fig. 3e and f that this short lockdown further reduced the current reproduction number and it levelled down the hospitalisation.
3. A relaxation of the measures during the festive period (5 December 2020-5 January 2020). This relaxation made the current reproduction number grow to well-above 1, which further led to a surge in infections and hospitalisations.
4. Another national lockdown (5 January 2021-12 March 2021). This second lockdown lasted for more than 2 months and brought the current reproduction number below 1. It was extremely effective in bringing down the infections and hospitalisations to a manageable level for the NHS.
5. A gradual lifting of the restrictions (12 March 2021-1 June 2021). While the relaxation of the measures culminated in an increase of the current reproduction number, thanks to the vaccination campaign, it led only to a surge in numbers of the infected population from June 2021, without a significant increase in hospitalisation and deaths^3^.

We undertake a retrospective test by considering the real data over the period from 1 October 2020 to 1 June 2021, and by applying the proposed model-driven intervention policy to keep the hospital bed occupancy below a threshold fixed at 20,000. As illustrated by Fig. 3, our SIHRD-V model-driven intervention policy (shown in Fig. 3e) is capable of maintaining the hospitalised population below the fixed threshold. Consequently, there would have been the potential to reduce the monthly average value of the hospitalisations by 58%. As shown in Fig. 3, the hospitalisation levels see a reduction even in the case when our policy is updated every 7 days or every 21 days. Though the fastest update (7 days) seems to offer the best performance, this has severe limitations in terms of practical implementation when considering the societal requirements. Our intervention policy, as shown in Fig. 3e, retrospectively prescribes the following interventions:

1. A national lockdown (0.88 level of restrictions) from November 2020 to February 2021, with a brief relaxation during December 2020, so as to bring the current reproduction number below 1 and to keep the hospitalisation below 20,000.
2. A recommendation to avoid the relaxation of the restrictions in December 2020. This would have prevented the surge in hospitalisation experienced in the UK in December 2020-January 2021.
3. The possibly to ease the restrictions from February 2021.

According to Table 1, the performance metric is equal to zero (i.e. the hospitalised population does not passes its threshold) if the period of the interventions is 7 days or 14 days. The metric is equal to 280 if the period is set equal to 21 days. Note, however, that the metric is equal to 1761 if the actual policy followed by the UK (model-free scenario) is considered, as the hospitalised population significantly passes the threshold in this scenario.

### Prospective test

The second key-finding of our study is focused on a potential future scenario for the period August 2021-May 2022. We consider two possible situations: (1) the spread of a Coronavirus Delta Variant, characterised by a basic reproduction number equal to 7^28^. (2) The surge of a new COVID-19 variant characterised by a basic reproduction number of 10, and by an hospitalisation rate 50% higher than the identified one. This variant surges from October 2021. Also, since there are still uncertainties around the actual duration of the immunity induced by the COVID-19 vaccine^36–39^, we assume that the immunity decays exponentially over time and we assess its impact on the pandemic evolution. We consider different values for the decay constant, specifically: 300, 800 and 1200 days.

Additionally, we adopt a time-varying threshold for the hospitalisations: it is equal to 20,000 in the period August-November 2021; it decreases to 10,000 in the period November 2021-January 2022; then it increases back to 20,000 from February 2022 onward. This threshold adjustment enables the NHS to better manage its capacity during the winter months. We compare again our model-driven intervention policy with a situation where the restrictions are not adjusted and they remain at the low level. Figs. 4 and  5 show the main findings of our predictive simulations. The lack of active intervention and complete relaxation of rules appear to be inefficient in dealing with the considered potential COVID-19 super-spreading event, as denoted in red in Figs. 4 and  5 . If a complete relaxation of the rules is adopted, our simulation predicts a peak of hospitalised population above 30,000 (Fig. 4) or above 40,000 if a new dangerous variant surges (Fig. 5). On the other hand, our intervention strategy recommends the following: (1): From Fig. 4e, as no new variant appears, the advice is to maintain an intermittent low level of restrictions. If the immunity decays faster within the population (the dashed green line situation depicted in Fig. 4e), a medium level of restrictions is necessary to be in place also during May 2022. (2): From the scenario depicted in Fig. 4e, as a new variant surges, an intermittent scheme of medium and low restrictions are necessary to be kept in place during the whole period. This would also prevent a further re-surge of infections and hospitalisation from March 2022 (black line in Fig. 5a,b) due to the immunity decay. According to Table 2, the performance metric reaches its maximum value if the mode-free scenario is adopted (independently of the possible occurrence of a new COVID-19 variant). As intuitively expected, the metric is equal to 0 if the the vaccine-induced immunity decays slower (scenario sihrdv-driv vd 1200). The metric increases if the immunity decay is faster, and also if new variants arise. Nonetheless, the benefits of our scheme compared to the model-free scenarios are also quantitatively demonstrated by Table 2

## Discussion

In this paper, we have shown that a periodic and automatic model-based algorithm can be used to maintain the number of people in hospital with COVID-19 under a chosen (and also time-varying) threshold. The potential benefits of our findings have been demonstrated considering both a retrospective and prospective analysis. As for the retrospective test, we have compared our policy with the one actually adopted in the UK for the period October 2020-June 2021. We have demonstrated the benefits of our proposal in terms of its capability of better mitigating the impact of the COVID-19 spread on the NHS. In particular, our model recommended that maintaining a lockdown through December 2020 would have led to an earlier relaxation of restrictions in February 2021.

For the future analysis, we have modelled possible surges of new COVID-19 variants, which are characterised by higher transmission and hospitalisations rates. Additionally, we have modelled the potential decay of the vaccination-driven immunisation within the population, which will lead to further spread of COVID-19, with the associated surge in the hospitalisation. In such scenarios, we have tested the behaviour of our periodic intervention policy compared to a model-free policy. We demonstrated the flexibility of our proposed scheme in offering guidance on the level of interventions which might be required, based on specific available information so far and certain expected scenarios, to maintain the hospitalised population below a time-varying threshold (to account for a seasonal pressure due to winter).

As in any other model-based study, our proposal is characterised by some limitations, which are expected to be addressed by the authors in future studies. In particular, despite the fact that our scheme accurately described the behaviour of the COVID-19 pandemic as demonstrated by Fig. 2, it does not model the spread of COVID-19 thorough the geographical regions of the UK. Furthermore, in the present study, the population has not been partitioned into age groups. These groups would better describe the impact of the Coronavirus spread on the hospitalised and deceased populations. The adopted approach in this paper does not explicitly account for the possible effects of multiple vaccine dosage administration or the impact of the prioritisation of the elderly and those at higher risk. Moreover, the different efficacies of the vaccines currently approved by the NHS in the UK, or elsewhere in the world has not been detailed, and an average value of the efficacy has been selected. The mathematical description of these aspects is expected to better mimic the real behaviour of the COVID-19 pandemic.

**Table 1:** The value of the Performance Metric ${\mathcal {E}}_H$ for the retrospective analysis.

	Sihrdv-driv 14d	Sihrdv-driv 7d	Sihrdv-driv 21d	Model-free
Performance Metric ${\mathcal {E}}_H$, Fig. 3	0	0	280	1761

**Table 2:** The value of the Performance Metric ${\mathcal {E}}_H$ for the retrospective tests.

	Sihrdv-driv vd800	Sihrdv-driv vd300	Sihrdv-driv vd1200	Model-free
Performance Metric ${\mathcal {E}}_H$, Fig. 4	0	85	0	310
Performance metric ${\mathcal {E}}_H$, Fig. 5	0	2597	0	4493

## Methods

### Epidemiological model

In this paper, we consider an extended version of the epidemiological compartmental SIHRD model^25^ to describe how each compartment of the population evolves over time. The developed model consists of the susceptible population *S*(*t*), the infected population *I*(*t*), the hospitalised population *H*(*t*), the recovered population *R*(*t*), the deceased population *D*(*t*) and the vaccinated population *V*(*t*), such that

1 $$\begin{aligned} N=S(t)+I(t)+H(t)+R(t)+D(t)+V(t) \end{aligned}$$

where *N* represents the total population. The model is denoted throughout this paper with the acronym SIHRD-V. It can be represented by the schematic in Fig. 1, and it is governed by the following set of equations:

2 $$\begin{aligned} {\dot{S}}(t)= & {} -\frac{\beta _{0}}{N}\left( 1-u(t)\right) I(t)S(t)\nonumber \\&-(1-f_{RS}(t)){\tilde{V}}(t) \end{aligned}$$

3 $$\begin{aligned} {\dot{I}}(t)= & {} \frac{\beta _{0}}{N}\left( 1-u(t)\right) I(t)S(t)-\left( \gamma +\lambda +\mu \right) I(t) \end{aligned}$$

4 $$\begin{aligned} {\dot{H}}(t)= & {} \lambda I(t)-\nu H(t) {-\mu _H H(t)} \end{aligned}$$

5 $$\begin{aligned} {\dot{R}}(t)= & {} \gamma I(t)+\nu H(t)-f_{RS}(t){\tilde{V}}(t) \end{aligned}$$

6 $$\begin{aligned} {\dot{D}}(t)= & {} \mu I(t){+\mu _H H(t)} \end{aligned}$$

7 $$\begin{aligned} {\dot{V}}(t)= & {} {\tilde{V}}(t) \end{aligned}$$

8 $$\begin{aligned} {\tilde{V}}(t)= & {} \alpha _V \big ( V_{\mathrm {min}} +f(u(t))\big ) \end{aligned}$$

9 $$\begin{aligned} y_1(t)= & {} H(t) \end{aligned}$$

10 $$\begin{aligned} y_2(t)= & {} \lambda I(t) \end{aligned}$$

The model parameters have the following meanings:

- $\beta _0$ is the transmission rate;
- $\lambda$ is the hospitalisation rate of the infected population;
- $\gamma$ is the recovery rate of the infected population;
- $\nu$ is the recovery rate of the hospitalised population;
- $\mu$ is the mortality rate of the infected population;
- $\mu _H$ is the mortality rate of the hospitalised population;
- $\alpha _V$ is the vaccine efficacy;
- $V_{min}$ is the minimum vaccination rate.

We consider the term *u*(*t*) as the authority intervention levels. The restrictions/lockdown control $0\le u(t) \le 1$, which is to be designed, is aimed at alleviating the rate of infections whilst keeping the hospitalisation at a manageable level. The signal *V*(*t*) accounts for the total number of people vaccinated up to time instant *t*, whilst the signal ${\tilde{V}}(t)$ represents the vaccination rate, which is the first time derivative of *V*(*t*). The signal *f*(*u*(*t*)) is the realisable vaccination policy adjustment. In contrast to existing approaches^13^, in which the vaccination roll-outs has been undertaken considering only the susceptible population, we assume to vaccinate the population belonging to both the susceptible *S*(*t*) and to the recovered *R*(*t*) compartments, which is true in practice^3^. Therefore, the dynamical model appropriately reflects this principle: the signal $f_{RS}(t)$ is defined as

11 $$\begin{aligned} f_{RS}(t):=\frac{R(t)}{S(t)} \end{aligned}$$

which represents the ratio between the recovered and the susceptible population. Given the vaccination rate ${\tilde{V}}(t)$, we assume to vaccine the fraction $(1-f_{RS}(t)){\tilde{V}}(t)$ from the susceptible population and the fraction $(f_{RS}(t)){\tilde{V}}(t)$ from the recovered population, as introduced in equations (2) and (5), respectively.

The measured quantities are indicated by $y_1(t)$ and $y_2(t)$, which are, respectively, the hospitalised population (*H*(*t*)), and the infected population being admitted to hospital at time *t* ($\lambda I(t)$). The two measurements are practically acquired on a daily basis via the NHS in the UK^3^. For a more compact representation of the model, the positive auxiliary constant

12 $$\begin{aligned} \varphi :=\gamma +\lambda +\mu \end{aligned}$$

can be introduced.

The Basic Reproduction Number, ${\mathcal {R}}_0$, is defined as the expected number of secondary infected cases caused by a single infected individual in a completely susceptible population^27^. ${\mathcal {R}}_0$ for the SIHRD-V model (2)-(10) can be defined as^27^

13 $$\begin{aligned} {\mathcal {R}}_0:=\frac{\beta _0}{\varphi } \end{aligned}$$

The Reproduction Number ${\mathcal {R}}(t)$, instead, is a time-varying quantity, and is defined as the average number of new infections (within a population that is only partially susceptible) caused by a single infected individual^27^. In order to derive an expression for ${\mathcal {R}}(t)$ we introduce the auxiliary signal $\beta (t)$ as

14 $$\begin{aligned} \beta (t):=\beta _0(1-u(t)) \end{aligned}$$

${\mathcal {R}}(t)$ for the SIHRD-V model (2)-(10) can be defined as^13^

15 $$\begin{aligned} {\mathcal {R}}(t):=\frac{\beta (t)}{\varphi } \end{aligned}$$

It is also possible to introduce an additional time-varying parameter, named the Current Reproduction Number ${\mathcal {R}}_c(t)$, which is defined as

16 $$\begin{aligned} {\mathcal {R}}_c(t):=\frac{{\mathcal {R}}(t) S(t)}{N} \end{aligned}$$

By making use of (14) and (15), it is possible to rewrite the model (2)-(10) as

17 $$\begin{aligned} {\dot{S}}(t)= & {} -\frac{{\mathcal {R}}(t)\varphi I(t) S(t)}{N}-(1-f_{RS}(t)){\tilde{V}}(t) \end{aligned}$$

18 $$\begin{aligned} {\dot{I}}(t)= & {} \frac{{\mathcal {R}}(t)\varphi I(t) S(t)}{N}- \varphi I(t) \end{aligned}$$

19 $$\begin{aligned} {\dot{H}}(t)= & {} \lambda I(t)-\nu H(t) {-\mu _H H(t)} \end{aligned}$$

20 $$\begin{aligned} {\dot{R}}(t)= & {} \gamma I(t)+\nu H(t)-f_{RS}(t){\tilde{V}}(t) \end{aligned}$$

21 $$\begin{aligned} {\dot{D}}(t)= & {} \mu I(t){+\mu _H H(t)} \end{aligned}$$

22 $$\begin{aligned} {\dot{V}}(t)= & {} {\tilde{V}}(t) \end{aligned}$$

23 $$\begin{aligned} {\tilde{V}}(t)= & {} \alpha _V \big ( V_{\mathrm {min}} +f(u(t))\big ) \end{aligned}$$

24 $$\begin{aligned} y_1(t)= & {} H(t) \end{aligned}$$

25 $$\begin{aligned} y_2(t)= & {} \lambda I(t) \end{aligned}$$

which is employed here for the model identification. If the Current Reproduction Number ${\mathcal {R}}_c(t)$ is considered, equations (17)-(18) can be rewritten as:

26 $$\begin{aligned} {\dot{S}}(t)= & {} -{\mathcal {R}}_c(t)\varphi I(t) -(1-f_{RS}(t)){\tilde{V}}(t) \end{aligned}$$

27 $$\begin{aligned} {\dot{I}}(t)=\; & {} \varphi \Big ({\mathcal {R}}_c(t)-1 \Big ) I(t) \end{aligned}$$

From (27), note that *I*(*t*) increases whenever ${\mathcal {R}}_c(t)>1$.

#### Epidemiological model identification

We identified the model parameters $\gamma ,\ \lambda , \ \nu , \ \mu$ relying on the official data^3^, which is available for the period 1 October 2021-1 June 2021, which accounts for the so-called second wave of COVID-19 in the United Kingdom. Specifically, we utilise the so-called nonlinear grey-box model identification approach^33^ based on the dynamics (17)-(25). The inputs to the identification algorithms are the official time-series $y_1(t)$ and $y_2(t)$, ${\mathcal {R}}(t)$, along with the time-series of the vaccination rate ${\tilde{V}}(t)$. We consider an average vaccine efficacy of $\alpha _V=0.9$^40^. The method determines the values of the parameters which minimise the square of the errors. The results of the identifications are summarised in Table 3. The study is in accordance with relevant guidelines and regulations. No human experiments have been undertaken. The only human-related data are the open data publicly available via the UK GOV source^3^.

**Table 3:** Identified parameters for the SIHRD-V model for the United Kingdom.

Symbol and meaning	Identified values
starting date	01 Oct 2020
ending date	01 Jun 2021
$\gamma$: recovery rate of the infected	0.1150
$\lambda$: hospitalisation rate	0.0103
$\mu$: mortality rate of the infected	0.0020
$\mu$: mortality rate of the hospitalised	0.0100
$\nu$: recovery rate of the hospitalised	0.0954

#### Immunity decay after vaccination

We assume that the the immunity gained via vaccination is exponentially decaying. This principle can be translated into the SIHRD-V model by rewriting the equations (2)-(10) as:

28 $$\begin{aligned} {\dot{S}}(t)= & {} -\frac{\beta _{0}}{N}\left( 1-u(t)\right) I(t)S(t) \nonumber \\&-(1-f_{RS}(t)){\tilde{V}}(t) + \varvec{\frac{1}{\tau _{v_d}} V(t)} \end{aligned}$$

29 $$\begin{aligned} {\dot{I}}(t)= & {} \frac{\beta _{0}}{N}\left( 1-u(t)\right) I(t)S(t)-\varphi I(t) \end{aligned}$$

30 $$\begin{aligned} {\dot{H}}(t)= & {} \lambda I(t)-\nu H(t) {-\mu _H H(t)} \end{aligned}$$

31 $$\begin{aligned} {\dot{R}}(t)= & {} \gamma I(t)+\nu H(t)-f_{RS}(t){\tilde{V}}(t) \end{aligned}$$

32 $$\begin{aligned} {\dot{D}}(t)= & {} \mu I(t) {+\mu _H H(t)} \end{aligned}$$

33 $$\begin{aligned} {\dot{V}}(t)= & {} {\tilde{V}}(t)-\varvec{\frac{1}{\tau _{v_d}} V(t)} \end{aligned}$$

34 $$\begin{aligned} {\tilde{V}}(t)= & {} \alpha _V \big ( V_{\mathrm {min}} +f(u(t))\big ) \end{aligned}$$

35 $$\begin{aligned} y_1(t)= & {} H(t) \end{aligned}$$

36 $$\begin{aligned} y_2(t)= & {} \lambda I(t) \end{aligned}$$

where the additional terms are in **bold**, and $\tau _{v_d}$ represents the exponential decay time constant. Ongoing studies and trials are being undertaken to evaluate the immunisation decay over time.

### Control approach

#### Sliding mode principle

Sliding Mode Control (SMC) techniques have been successfully proposed in the existing literature for robust control of nonlinear uncertain systems^41^. SMC schemes enforce the trajectories of the controlled system to slide along a so-called hyper-surface giving rise to a system behaviour named sliding mode. The hyper-surface is defined as a function of the states of the controlled system to be nullified in finite time, thus solving the required control problem^42,43^. In particular, SMC can be used to track a specific reference value or to satisfy specific constraints on the system to ensure safety and reliability. SMC has been proven to enforce finite time stabilisation of the controlled system, whilst rejecting bounded uncertainties and disturbances appearing in the input channel of the systems^41^. In order to select the appropriate SMC method, the relative degree of the hyper-surface with respect to the control input has to be known. If the control signal explicitly appears for the first time in the *r*-th time derivative of the hyper-surface, then the number *r* is called the relative degree^44^. Amongst the existing SMC approaches, the so-called Sub Optimal Sliding Mode (SOSM) was originally proposed in the nineties^45^. SOSM has been revealed to be easy to implement in experimental setups. Furthermore, SOSM can be effectively used for systems with relative degree one to avoid the application of discontinuous control action^45^. In the present paper, we make use of a particular version of the SOSM method originally proposed by one of the authors of this manuscript^46^.

#### Control design

The fundamental short term goal of the authorities is to maintain the hospitalised population *H*(*t*) under a threshold that is a-priory imposed due to the bed capacity constraints of the NHS. With this in mind, we select the hyper-surface for the SMC such that the solution of the mathematical model attains the desired goal when the evolution is confined to the proposed hyper-surface. Towards this end, suitable feedback policies are devised so that the solution reaches and stays on the hyper-surface (or manifold) and renders this set forward invariant. Consequently, the goal of maintaining the hospitalised population *H*(*t*) below the prescribed threshold is achieved. By virtue of the theoretical features of the SMC design, the goal achievement occurs with certain level of robustness against a specific class of perturbations which may appear in realistic operating scenarios.

##### Assumption 1

(for Control Design) It is assumed that:

1. The susceptible population *S*(*t*) and the infected population *I*(*t*) are always strictly greater than zero, i.e. $S(t)>0, \ I(t)>0$.
2. At any fixed time instant *t*, the hospitalised population can always be written as a fraction of the infected population, i.e.
  37 $$\begin{aligned} H(t)=\eta (t) I(t) \end{aligned}$$
  where $\eta (t)$ is an unknown positive time-varying parameter satisfying $0<\eta (t)<1$.
3. Herd immunity is not achieved yet, which means that the auxiliary variable $\delta (t)$ satisfies the following
  38 $$\begin{aligned} \delta (t):=\frac{S(t)}{N}, \ \ 1/2<\delta (t)<1 \end{aligned}$$
4. For the control design, the model parameters, which have been identified relying on the COVID-19 data^3^, are assumed positive but unknown with known bounds.

In the control approach, the objective is to have

39 $$\begin{aligned} H(t) \le H_{\mathrm {max}} \end{aligned}$$

where $H_{\mathrm {max}}$ is known and defined a priori. In practice, $H_{\mathrm {max}}$ represents the population that can be hospitalised without overwhelming the NHS. The hyper-surface, denoted as $\sigma (t)$ is selected as follows:

40 $$\begin{aligned} \sigma (t):=y_1(t)-H_{\text{ max }}+ a_H{y}_2(t) \end{aligned}$$

where $a_H$ is a positive design parameter. The selected hyper-surface $\sigma (t)$ is a linear combination of the hospitalised population $y_1(t)=H(t)$, of the infected population being admitted to hospital $y_2(t)=\lambda I(t)$, and of $H_{\mathrm {max}}$. Therefore, when the sliding mode is enforced, i.e. when the hyper-surface is equal to zero, one has

41 $$\begin{aligned} \sigma (t)=0 \rightarrow y_1(t)=H(t)=H_{\text{ max }}-\lambda a_H {I}(t) \end{aligned}$$

which guarantees that $H(t) \le H_{\text{ max }}$.

##### Remark 1

The proposed sliding variable is easy to compute in practice. Specifically, it is a function of the hospitalised population *H*(*t*) and of the infected population being admitted to hospital at time *t* ($\lambda I(t)$). These two quantities are typically known on a daily basis via the NHS records^3^. In contrast, existing approaches^25,31^ rely solely on the data concerning the infected population *I*(*t*), which is more difficult to measure^34^ and often underestimated^35^.

The proposed control approach is governed by:

42 $$\begin{aligned} \sigma (t)=H(t)-H_{\text{ max }}+ a_H\lambda I(t) \end{aligned}$$

43 $$\begin{aligned} u(t)=u(0)+\int _0^t w(\tau ) d\tau \end{aligned}$$

44 $$\begin{aligned} w(t)={\left\{ \begin{array}{ll} \alpha W \text{ sign }\Big (\sigma (t)-\frac{\sigma _{\star }}{2}\Big ) &{} \mathrm {if} \ \ 0<{u}(t)<1 \\ -W \text{ sign }\Big (u(t) \Big ) &{} \mathrm {if} \ u(t)\le 0 \\ &{} \text{ or } \ u(t) \ge 1 \ \end{array}\right. } \end{aligned}$$

where *u*(0) is the intervention policy at the initial condition, ($0\le u(0)\le 1$), $\alpha$ and *W* are positive design constants. Tuning rules for the same will be derived in the sequel of the paper. Note that in practice $\alpha$ and *W* determine how fast the policy update *u*(*t*) can occur, i.e. the higher the value, the faster the increase or decrease of the control *u*(*t*). The signal *w*(*t*) is an auxiliary variable, the function $\text{ sign }(\cdot )$ denotes the sign function, and $\sigma _\star$ is the value of the hyper-surface $\sigma (t)$ at the last time instant when the condition ${\dot{\sigma }}(t)=0$ was verified^45^. The vaccination policy is adjusted relying on the current restrictions imposed by the signal *u*(*t*). In particular, we consider increasing the vaccination rate whenever the restrictions are increased, in order to better alleviate the pressures on the NHS. Therefore:

45 $$\begin{aligned} {\tilde{V}}(t) = \alpha _V\big ( V_{\mathrm {min}} +k_Vu(t)\big ) \end{aligned}$$

As $0\le u(t) \le 1$, the positive design constant $k_V$ is tuned in such a way that ${\tilde{V}}_{\mathrm {max}}=V_{\mathrm {min}}+k_V$, where $V_{\mathrm {max}}$ denotes the maximum vaccination rate.

#### Stability analysis

In order to show that the control scheme governed by (42)–(45) ensures the key-inequality (39), we determine the first time derivative of $\sigma (t)$ which yields:

46 $$\begin{aligned} {\dot{\sigma }}(t)= {\dot{H}}(t)+ a_H \lambda {\dot{I}}(t) \end{aligned}$$

Given the SIHRD-V dynamical model (2)–(10), it follows that ${\dot{\sigma }}(t)$ can be written as

47 $$\begin{aligned} {\dot{\sigma }}(t)= & {} \lambda I(t) -\nu H(t) {-\mu _H H(t)} \nonumber \\&+a_H \lambda \Big [ \frac{\beta _{0}}{N}\left( 1-u(t)\right) I(t)S(t)-\varphi I(t) \Big ] \end{aligned}$$

After standard algebraic simplifications,

48 $$\begin{aligned} {\dot{\sigma }}(t)= & {} (\lambda -\varphi ) I(t) -\nu H(t) {-\mu _H H(t)} \nonumber \\&+ a_H \lambda \rho \frac{\beta _0}{N} I(t) S(t) -a_H \lambda \frac{\beta _0}{N} I(t)S(t)u(t) \end{aligned}$$

It can be noted that the relative degree of the sliding variable $\sigma (t)$ with respect to the control input *u*(*t*) is equal to one, as the control signal explicitly appears in (48). Furthermore, ${\dot{\sigma }}(t)$ can be compactly rewritten as

49 $$\begin{aligned} {\dot{\sigma }}(t)= g(t)+b(t)-b(t){u}(t) \end{aligned}$$

50a $$\begin{aligned} g(t):= & {} (\lambda -\varphi ) I(t) -\nu H(t) {-\mu _H H(t)} \end{aligned}$$

50b $$\begin{aligned} b(t):= & {} a_H \lambda \frac{\beta _0}{N} I(t)S(t) \end{aligned}$$

#### SIHRD-V dynamics during the sliding mode

We study the dynamical behaviour of the adopted SIHRD-V model during the the sliding mode, which is characterised by the conditions $\sigma (t)={\dot{\sigma }}(t)=0$. As ${\dot{\sigma }}(t)=g(t)+b(t)-b(t)u(t)$, the so-called equivalent control $u_e(t)$ is the specific control input that guarantees $0=g(t)+b(t)-b(t)u(t)$, which implies that

51 $$\begin{aligned} u_e(t)=1+\frac{g(t)}{b(t)}= 1+ \frac{(\lambda -\varphi ) I(t) -\nu H(t) {-\mu _H H(t)}}{ a_H \lambda \frac{\beta _0}{N} I(t)S(t)} \end{aligned}$$

We want to determine the tuning rules for $a_H$ such that it is guaranteed that

52 $$\begin{aligned} 0\le u_e(t) \le 1. \end{aligned}$$

Clearly, $g(t)<0$ as $\lambda <\varphi$ by definition (see equation (12)). It follows that $u_e(t)<1$. In order to ensure that $u_e(t)>0$, we need to impose the constraint $b(t)>-g(t)$, which yields

53 $$\begin{aligned} a_H \lambda \frac{\beta _0}{N} I(t)S(t)>(\varphi -\lambda ) I(t) +\nu H(t) {+\mu _H H(t)} \end{aligned}$$

Under Assumption 1, equation (53) can be rewritten as follows

54 $$\begin{aligned} a_H \lambda \beta _0 I(t)\delta (t)>(\varphi -\lambda +\eta (t)) I(t) \end{aligned}$$

Solving for $a_H$ yields

55 $$\begin{aligned} a_H>\frac{\varphi -\lambda +\eta (t)}{\lambda \delta (t)} \end{aligned}$$

Therefore, the value of $a_H$ should always be chosen to verify (55).
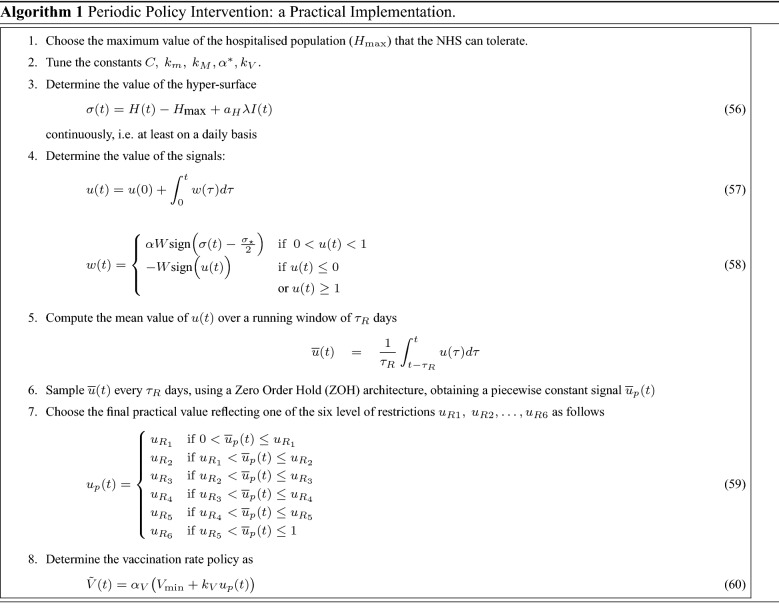


#### The dynamic evolution of the hyper-surface

In order to analyse the stability of the SMC, the so-called auxiliary system is introduced, which is composed of the hyper-surface $\sigma (t)$ and its first time derivative ${\dot{\sigma }}(t)$. By defining $x_1(t):=\sigma (t)$, $x_2(t):={\dot{\sigma }}(t)$, and given the control scheme in (42)–(45), the auxiliary system is governed by the following differential equations:

61a $$\begin{aligned} {\dot{x}}_1(t)= & {} g(t)+b(t)-b(t)u(t) \nonumber \\= & {} x_2(t) \end{aligned}$$

61b $$\begin{aligned} {\dot{x}}_2(t)= & {} {\dot{g}}(t)+{\dot{b}}(t)-{\dot{b}}(t)u(t)+ b(t)w(t) \nonumber \\= & {} h(t)+b(t)w(t) \end{aligned}$$

where

61c $$\begin{aligned} h(t):= & {} {\dot{g}}(t)+{\dot{b}}(t)-{\dot{b}}(t)u(t) \nonumber \\= & {} (\lambda -\varphi ){\dot{I}}(t)-\nu {\dot{H}}(t) {-\mu _H {\dot{H}}(t)}\nonumber \\&+a_H \lambda \frac{\beta _0}{N} \Big ( {\dot{I}}(t)S(t)+ I(t)\dot{{S}}(t)\Big ) \nonumber \\&- a_H \lambda \frac{\beta _0}{N} \Big ( {\dot{I}}(t)S(t)+ I(t)\dot{{S}}(t)\Big ) u(t) \end{aligned}$$

An expanded expression for *h*(*t*) can be determined utilising the model (2)–(10). Under Assumption 1, it is immediately clear that *h*(*t*) is bounded, as it is function of the first time derivative of the susceptible, infected and hospitalised populations. Given the maximum possible value of $|{\dot{S}}(t)|$, $|{\dot{I}}(t)|$ and $|{\dot{H}}(t)|$, it is possible to chose the the upper-bound *C* for *h*(*t*) such that $|h(t)|<C$. Furthermore, under Assumption 1, it follows that $k_m<b(t)<k_M$, where the positive constants $k_m$ and $k_M$ are chosen considering the possible minimum and maximum value of the infected and the susceptible populations.

The parameter $\alpha$ appearing in (44) is governed by^45,46^

62 $$\begin{aligned} \alpha ={\left\{ \begin{array}{ll} \alpha ^{*}\in (0,1]\cap (0,\frac{3k_{m}}{k_{M}}) &{} \mathrm {if}\ \left( \sigma (t)-\frac{\sigma _{\star }}{2}\right) \\ &{} \cdot \left( \sigma _{\star }-\sigma (t)\right) >0 \\ 1 &{} \mathrm {else} \end{array}\right. } \end{aligned}$$

The positive constant *W* satisfies

63 $$\begin{aligned} W>\max \left( \frac{C}{\alpha ^{\star }k_{m}},\ \frac{4C}{3k_{m}-\alpha ^{*}k_{M}}\right) \end{aligned}$$

Note that, once the values for *C*, $k_m$, and $k_M$ are chosen, the values for $\alpha$ and *W* in (62) and (63) can be computed accordingly. As proven in Theorem 1 and in Theorem 2 reported in^46^, given the control scheme, if $\alpha$ and *W* satisfy the conditions (62)-(63), the auxiliary system in (61a)-(61b) converges to the origin in finite time, guaranteeing that the condition (39) is satisfied in finite time.

#### Practical implementation of the control strategy

In practice, the signals *u*(*t*) and ${\tilde{V}}$ in (42)–(45) cannot be continuously updated on a daily basis, but only every $\tau _R$ days (e.g. every week or every two weeks). Furthermore, the signal *u*(*t*) should reflect specific restrictions in a wide range of applications, such as different rules in place for social mixing, opening of non-essential shops, retails, pubs and restaurants, and travelling throughout the country or abroad^19^. Therefore, in practice, *u*(*t*) cannot assume any possible value between 0 and 1. We postulate six possible practical values of the control signal relying on its actual history in the UK over the period October 2020-June 2021. We also match its changes with the policy adjustments dates (labelled as $1,\dots ,5$ in Fig. 3-e) to derive the corresponding descriptions of the social restrictions to be imposed. The chosen values and the associated rules are:

1. $\mathbf {u_{R1}=0.66}$: **No restrictions in place** other than the recommendations about crowded places and personal hygiene.
2. $\mathbf {u_{R2}=0.77}$: **Low level of restrictions**, with certain limits on large events and social gatherings.
3. $\mathbf {u_{R3}=0.82}$: **Medium level of restrictions** with limits on indoor social gatherings
4. $\mathbf {u_{R4}=0.84}$: **Enhanced level of restriction**, with no indoor social gathering.
5. $\mathbf {u_{R5}=0.86}$: **High level of restrictions**, with closures of all hospitality sectors
6. $\mathbf {u_{R6}=0.88}$: **National lockdown**, with closure of all non-essential shops and stay-at-home instruction.

Consequently, the practical implementation of our novel periodic intervention policies can be summarised by the pseudo-code in Algorithm 1.

### Performance metric for the control strategy

We define the following performance metric to evaluate the effectiveness of our control strategy on the hospitalised population *H*(*t*):

64 $$\begin{aligned} {{e}_H(t):={\left\{ \begin{array}{ll}H(t)-H_{{max}} &{} {if H(t)-H_{{max}}>0} \\ 0 &{} {else} \end{array}\right. }} \ \ \ \ \ \ \ {{\mathcal {E}}_H(t):=\dfrac{1}{t}\int _0^t e_H(s) ds, \ \ \ \ {\mathcal {E}}_H(0)=0}, \end{aligned}$$

where $e_H(t)$ represents the excess of hospitalised population *H*(*t*) with respect to the threshold $H_\text {max}$ (and it is equal to 0 if the hospitalised population remains below the threshold), whilst ${\mathcal {E}}_H(t)$ is the mean value of $e_H(t)$ over the time period [0, *t*] (days).
